# Long-Term Protection against Virulent Newcastle Disease Virus (NDV) in Chickens Immunized with a Single Dose of Recombinant Turkey Herpesvirus Expressing NDV F Protein

**DOI:** 10.3390/vaccines12060604

**Published:** 2024-05-31

**Authors:** Bin Shi, Guifu Yang, Yue Xiao, Kun Qian, Hongxia Shao, Moru Xu, Aijian Qin

**Affiliations:** 1 Ministry of Education Key Laboratory for Avian Preventive Medicine, Yangzhou University, No.12 East Wenhui Road, Yangzhou 225009, China; 2 Jiangsu Co-Innovation Center for Prevention and Control of Important Animal Infectious Diseases and Zoonoses, No.12 East Wenhui Road, Yangzhou 225009, China

**Keywords:** Newcastle disease virus, recombinant turkey herpesvirus, insertion site, humoral immunity, long-term protection

## Abstract

Newcastle disease (ND) is a significant infectious disease in poultry, causing substantial economic losses in developing countries. To control ND, chickens must be vaccinated multiple times a year. In order to develop an improved vaccine that provides long-term protection, the F gene from genotype VII NDV was inserted into the herpesvirus of turkey (HVT) vaccine virus using CRISPR/Cas9-mediated NHEJ repair and Cre/LoxP technology. The immunogenicity and protective efficacy of the resulting recombinant vaccines were evaluated through antibody assays and virus challenge experiments. Two recombinant vaccines, rHVT-005/006-F and rHVT-US2-F, were generated, both exhibiting growth rates comparable with those of HVT in vitro and consistently expressing the F protein. One-day-old specific pathogen-free (SPF) chickens immunized with 2000 PFU/bird of either rHVT-005/006-F or rHVT-US2-F developed robust humoral immunity and were completely protected against challenge with the NDV F48E8 strain at 4 weeks post-vaccination (wpv). Furthermore, a single dose of these vaccines provided sustained protection for at least 52 wpv. Our study identifies rHVT-005/006-F and rHVT-US2-F as promising ND vaccine candidates, offering long-term protection with a single administration. Moreover, HVT-005/006 demonstrates promise for accommodating additional foreign genes, facilitating the construction of multiplex vaccines.

## 1. Introduction

Newcastle disease (ND), caused by the virulent Newcastle disease virus (NDV), is widespread and affects numerous avian species, leading to significant losses in the poultry industry [1]. NDV, a member of the genus Orthoavulavirus [2] primarily produces six structural proteins, including the nucleoprotein (NP), the phosphoprotein (P), the matrix protein (M), the fusion protein (F), the hemagglutinin-neuraminidase (HN), and the large protein (L) [3,4]. Among them, the F and HN proteins are critical for virus attachment and infectivity [5], serving as the major protective antigens of NDV. Notably, the F protein has been reported to exhibit superior immunogenicity compared with the HN protein [6].

NDV strains are categorized into two clades, class I and class II, within a single serotype [7]. Class I NDVs are almost avirulent. Class II NDVs are divided into 20 genotypes (I–XXI) [8], most of which are highly virulent based on the F gene cleavage site. Genotype VII NDV has recently become the prevalent strain in developing countries, with other virulent NDVs of genotypes VIII, IX, and XII also being sporadically reported [8,9]. Current NDV control relies heavily on vaccination, predominantly utilizing live and inactivated genotype I and/or II NDV vaccines [10]. Despite vaccination efforts, ND continues to affect the global poultry industry, causing regional outbreaks and significant economic losses. In order to achieve a good protective effect, multiple boosters were required in the current immunization program, increasing the stress in the flock.

Compared with traditional live and inactivated ND vaccines, herpesvirus of turkey (HVT) is an ideal viral vector for developing vaccines due to its non-pathogenic nature and ability to induce a lifelong immune response in chickens with a single dose of immunization [11]. HVT has been successfully employed in the development of vector vaccines to combat various avian diseases, including infectious bursal disease (IBD) [12,13], infectious laryngotracheitis (ILT) [14], avian influenza (AI) [15,16], and ND [17,18,19]. The commercialization and global application of HVT-IBD vaccines, particularly with the Vaxxitek HVT-IBD vaccine, have garnered considerable attention. This vaccine is especially popular in several countries, including China [20,21], due to its exemplary safety profile [20], early protection capabilities, and resistance to maternal antibodies [21]. Meanwhile, the international market has introduced several high-quality HVT ND vaccines, such as Vectormune ND [18] and Innovax ND [22], providing powerful tools for controlling ND. However, due to the antigenic differences between the F proteins expressed by these vaccines (originating from genotypes I and II) and the currently prevalent virulent strains (such as genotypes VII, VIII, IX, or XII) [23,24], researchers have begun to focus on the development of recombinant HVT ND vaccines that express the F protein of NDV virulent strains. For instance, one vaccine expressing the genotype VII NDV F protein at the US2 site has demonstrated a 70% protection rate against a homologous NDV challenge five weeks post-vaccination (wpv) [25]. Another vaccine, expressing the genotype XII F protein at the UL45/UL46 site [26], has shown validated protective efficacy at 7 wpv; their early protection capabilities need further evaluation, and the long-term protective effects of both vaccines still require investigation.

The selection of protective antigens and determination of insertion sites are crucial for the efficacy of recombinant vaccines [6,15,27]. A stable new insertion site, HVT-005/006 [28], has been previously identified, but its potential to express the protective antigen of NDV deserves further evaluation. Additionally, it has been reported that DNA vaccines expressing artificially modified virulent NDV F protein (with its cleavage site mutated to an avirulent strain) have exhibited superior immunogenicity compared with those expressing the wild-type F protein [29]. Therefore, this study aims to compare the US2 site with the newly discovered HVT-005/006 site by inserting the artificially modified genotype VII NDV F protein to develop recombinant HVT vaccines, and to comprehensively evaluate their long-term protective efficacies against ND. Through this research, we hope to develop an HVT-ND vaccine that offers protection for a longer time. Considering the frequent occurrence of multiple pathogens and the prevalence of co-infections in poultry [30], HVT vector vaccines capable of preventing multiple pathogens by co-expressing several antigens have become a research focus [13,31]. Therefore, discovering new, stable, and enduring expression sites for foreign genes in the HVT virus vector is crucial. Through this research, we also aim to validate whether the HVT-005/006 site can serve as a new site for developing multivalent HVT vector vaccines. By establishing this site as a viable option for stable antigen expression, we hope to broaden the use of the HVT vector to prevent various pathogens simultaneously.

## 2. Materials and Methods

### 2.1. Animals, Viruses, and Cell Culture

Nine-day-old chicken embryos and one-day-old SPF White Leghorn chickens were obtained from Beijing Merial Vital Laboratory Animal Technology Co., Ltd., Beijing, China.

HVT and recombinant HVT viruses were propagated in primary chicken embryo fibroblast (CEF) cells, which were maintained in Medium 199 (M199) supplemented with 5% fetal bovine serum (FBS), 100 U/mL penicillin, 100 μg/mL streptomycin and 10% tryptose phosphate broth at 38.5 °C under a 5% CO_2_ atmosphere. The NDV strain F48E8 was grown in 9-day-old specific pathogen-free (SPF) embryonated chicken eggs, with virus titers calculated as half embryo lethal doses (ELD_50_).

### 2.2. Construction of sgRNAs, Donor Plasmids, and Recombinant Viruses

The sg-US2-1, sg-HVT005/006-2, and sg-A sequences, as reported previously [28] (Table 1), were cloned into CRISPR/Cas9 vector PX330 using BbsI cloning sites. The F gene, derived from the genotype VII NDV strain and mutated at the cleavage site to GGQGRF, was cloned into the pcDNA-sfi vector via NotI sites to generate the plasmid pcDNA-sfi-F (Table 1). The F expression cassette was released from pcDNAsfi-F with restriction enzyme sfiI and cloned into yjr pGEM-sgA-LoxP-eGFP vector via sfiI sites, generating donor plasmid pGEM-sgA-LoxP-eGFP-F.

CEF cells were transfected with 0.25 µg PX330-sg-US2-1 or PX330-005/006-2, 0.25 µg PX330-sgA, and 0.25 µg donor plasmids per well of a 24-well plate. The CEF cells were infected with HVT at 0.01 plaque-forming units (PFUs)/cell at 12 h post-transfection. The virus was digested and propagated at 3 days post-transfection, and the recombinant virus was purified by the limiting dilution until all plaques exhibited fluorescence. To knock out the eGFP gene cassette using the Cre/LoxP system, 500 ng pcDNA3.1-Cre plasmid was transfected into CEF cells in a 24-well plate. 12 h after transfection, cells were infected with 100–200 PFU of rHVT-US2-eGFP-F or rHVT-005/006-eGFP-F. After 3 days, when a cytopathic effect was evident, the eGFP-negative plaque was picked and used to infect CEF cells in 24-well plates for further purification. PCR confirmation of the eGFP cassette knockout was performed by using primers for HVT-US2-F+R or HVT-005/006-F+R (Table 1).

### 2.3. Immunofluorescence and Western Blot

CEF cells grown on 24-well plates were infected with HVT, HVT-005/006-F, or HVT-US2-F at a multiplicity of infection (MOI) of 0.001 for 72 h. The cells were washed with PBS, fixed with 4% paraformaldehyde in PBS for 20 min, and permeabilized with 0.25% Triton-X in PBS for 10 min at room temperature. Subsequently, the cells were blocked with 1% bovine serum albumin in PBS and incubated for 1 h at room temperature with a monoclonal antibody (Mab) against HVT gB [28] (BD8, 1:400 dilution) and NDV-positive chicken serum (1:800 dilution) in PBS. Cells were washed with PBS and incubated for 1 h at room temperature with goat anti-mouse Alexa 488 MAb (1:800 dilution, Jackson Immunoresearch, West Grove, PA, USA) or goat anti-chicken Alexa Cy3 Mab (1:800 dilution, Jackson Immunoresearch). The images were captured using the OLYMPUS inverted fluorescent microscope, Olympus, Tokyo, Japan.

For the Western blot, CEF cells infected with HVT, rHVT-005/006-F, or rHVT-US2-F at MOI 0.01 for 72 h were lyzed using RIPA buffer. NDV-positive chicken serum (1:800) and MAb BD8 (1:400) were used to detect the F and HVT-gB proteins, respectively. HRP-conjugated goat anti-chicken IgY or anti-mouse IgG antibodies (1 in 10,000 dilutions, Jackson Immunoresearch) were visualized using a FluorChemE imaging system (Protein Simple, San Jose, CA, USA).

### 2.4. Stability and Growth Properties of the Recombinant Viruses

To assess the genetic stability of rHVT-US2-F and rHVT-005/006-F, each virus was serially passaged up to 15 times (P1–P15) in CEF cells. Every fifth passage, viral genomic DNA was extracted to confirm the presence and integrity of the inserted F gene via PCR, using specific primers, HVT-US2-F+R or HVT-005/006-F+R (Table 1). The size of the PCR products was analyzed to verify the correct insertion of the F gene. The expression of the F protein was detected via both immunofluorescence and the Western blot, as described above.

### 2.5. Animal Experiments

Forty-two 1-day-old SPF White Leghorn chickens were randomly divided into four groups, with thirteen chickens per group. Chickens in group 1 and group 2 were inoculated with 2000 PFU/bird of recombinant virus rHVT-US2-F or rHVT-005/006-F subcutaneously, while chickens in group 3 and group 4 were inoculated with an equal amount of dilution medium. At 4 wpv, chickens in Groups 1–3 were injected intramuscularly with the 10^4^ELD_50_ NDV F48E8 strain. Chickens in Group 4 were injected with the dilution buffer. At three days post-challenge (3 dpc), three chickens from each group were euthanized. The spleen, proventriculus, and cecum tonsils were collected for lesion observation and histopathological examination. Additionally, samples of these three organs were also collected to determine the NDV viral load. The collected tissue organs were homogenized and subjected to three freeze–thaw cycles, followed by centrifugation at 8000 rpm for 5 min at 4 °C to collect the supernatant. DF1 cells were used to determine the TCID_50_ of the virus. Other chickens were continuously observed for 14 days after the challenge, and we recorded the results on morbidity and mortality.

To assess the long-term protective efficacy of the recombinant virus, a second animal experiment was performed in the above groups. These birds were evaluated as mentioned above at 12, 20, and 52 wpv.

### 2.6. Serological Tests

To measure serum-specific IgY antibodies against NDV F antigen, serum samples were obtained from the birds every 7 days post-vaccination. In a second animal experiment, sera were collected at 12 wpv and then every 8 weeks. Antibody titers of the sera were determined using a commercial ELISA antibody kit (IDvet, Deauville, France) following the manufacturer’s instructions (titers ≥993 were considered positive). To determine the neutralizing antibody titers against NDV in the serum [32], a two-fold serial dilution of 50 μL of heat-inactivated serum was performed. The serum was then incubated with 100 TCID_50_/50 μL of NDV F48E8 virus at room temperature for 1 h. The mixture was transferred to a 96-well culture plate containing DF1 cells. After 72 h of incubation, a hemagglutination assay was employed to ascertain the quantity of infected cells. The neutralization titer represents the highest diluted antibody preventing the viral infection of cells.

### 2.7. Detection of Viral Shedding

The oropharyngeal swabs and cloacal swabs were collected at days 3, 5, 7, and 10 post-challenge, and then inoculated in 9-day-old SPF chicken embryos after treatment. The non-specific chicken embryos that died within 24 h were removed, and the remaining embryos were examined every 12 h. The dead chicken embryos were refrigerated in a 4 °C refrigerator overnight to collect the allantoic fluid, and the chicken embryos that survived after 96 h were placed in a 4 °C refrigerator overnight to collect the allantoic fluid. Virus presence in allantoic fluid was determined using the HA test, with an HA titer ≥3 considered NDV-positive.

### 2.8. Statistical Analysis

All statistical analyses were performed using GraphPad Prism v8.0.2 (GraphPad Software, San Diego, CA, USA). Differences in replication rates between different recombinant viruses and HVT were compared via one-way ANOVA, as were differences in antibody titers between the inoculated and uninoculated groups. Differences were considered significant at * *p* < 0.05, ** *p* < 0.01, *** *p* < 0.001, and **** *p* < 0.0001.

## 3. Results

### 3.1. Construction of rHVT-005/006-F and rHVT-US2-F

**Figure 1:**
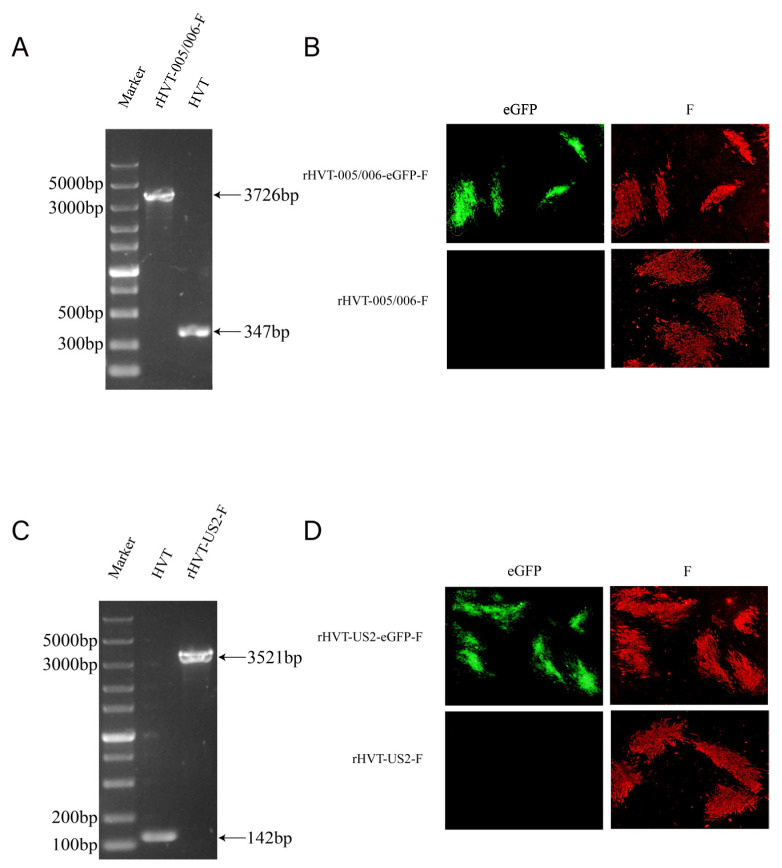
Construction and verification of rHVT-US2-F and rHVT-005/006-F. (**A**) PCR identification of rHVT-005/006-F. (**B**) CEF infected with rHVT-005/006-eGFP-F or rHVT-005/006-F identified via IFA (40×). (**C**) PCR identification of rHVT-US2-F. (**D**) CEF infected with rHVT-US2-eGFP-F or rHVT-US2-F identified via IFA (40×).

The NDV F gene expression cassettes were inserted into the HVT-US2 or HVT-005/006 sites using CRISPR/Cas9-mediated non-homologous end joining (NHEJ) and Cre/LoxP recombination systems (Figures S1 and S2). CEF cells were co-transfected with CRISPR Cas9/gRNA plasmids and donor plasmids, then infected with HVT viruses. Two strains of recombinant viruses co-expressing the eGFP and F gene were obtained, named rHVT-US2-eGFP-F or rHVT-005/006-eGFP-F (Figure 1B,D). Subsequently, the eGFP gene cassette was excised by the Cre/LoxP system, which was achieved by transfecting it with the pcDNA3.1-Cre plasmid into CEF cells infected with rHVT-US2-eGFP-F and rHVT-005/006-eGFP-F viruses. rHVT-US2-F and rHVT-005/006-F were obtained after several rounds of purification. PCR and IFA results confirmed the expected sizes of the PCR products and the expression of the F protein by the two recombinant viruses (Figure 1A–D).

### 3.2. Characterization of the rHVT-005/006-F and rHVT-US2-F In Vitro

**Figure 2:**
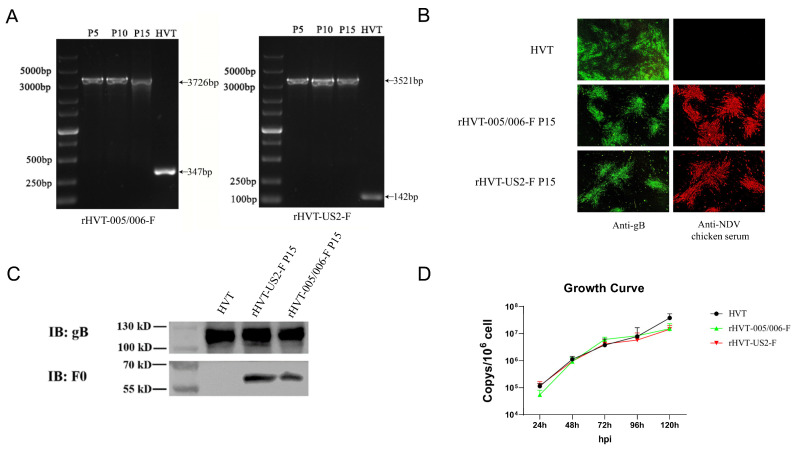
Characterization of rHVT-US2-F and rHVT-005/006-F in vitro. (**A**) Stability assessment of the F cassette in rHVT-005/006-F and rHVT-US2-US2-F via PCR. (**B**) CEF cells infected with the 15th passage of rHVT-005/006-F or rHVT-US2-F, as well as HVT, identified by IFA. (**C**) Western blot analysis of rHVT-05/06-F or rHVT-US2-F and HVT. (**D**) Growth kinetics of rHVT-005/006-F, rHVT-US2-F and HVT in vitro. The y-axis represents the viral copy number per 1,000,000 cells.

To evaluate the viral gene stability, CEF cells were infected with HVT, rHVT-005/006-F, or rHVT-US2-F, respectively, for 15 continuous passages. Viral DNA was extracted every fifth passage for PCR analysis. The results demonstrated that rHVT-005/006-F showed only a 3726 bp band, while the HVT showed a 347 bp band (Figure 2A); rHVT-US2-F showed only a 3521 bp band, while HVT showed only a 142 bp band (Figure 2A), indicating the stability of the inserted fragments. Cells infected with these recombinant viruses at the 15th passage were examined via IFA using MAb BD8 to HVT gB and NDV-positive chicken serum as primary antibodies. The results confirmed that both rHVT-005/006-F and rHVT-US2-F stably expressed F protein in CEF cells (Figure 2B). Similarly, F protein expression was recognized by NDV-positive chicken serum in Western blot analyses. (Figure 2C).

To assess whether or not the insertion of the F gene affects viral replication, CEF cells in six-well plates were infected with 100PFU of HVT, rHVT-US2-F, or rHVT-005/006-F, respectively. Viral replication rates, measured via qPCR, showed no significant differences between the recombinant and parental HVT virus (Figure 2D).

### 3.3. Humoral Immune Response to Vaccination in SPF Chickens

**Figure 3:**
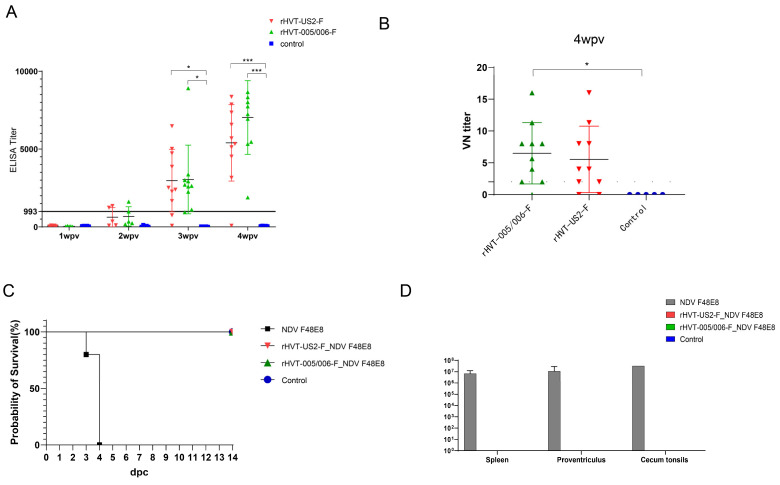
(**A**) rHVT-005/006-F- or rHVT-US2-F-induced humoral response. The y-axis represents antibody titers. Titers ≥933 were considered positive. The x-axis represents serum collected at 1, 2, 3, and 4 weeks post-vaccination (wpv). (**B**) Neutralizing antibody titers against virulent NDV in SPF chickens immunized with rHVT-005/006-F or rHVT-US2-F at 4 wpv. (**C**) Survival curves of chickens immunized with rHVT-005/006-F or rHVT-US2-F challenged with virulent NDV. (**D**) NDV viral load in different organs after challenge with virulent NDV. * *p* < 0.05, *** *p* < 0.001.

The immunogenicity of the rHVT-F vaccine was evaluated by collecting serum samples from each group and determining antibody titers against the F protein using an ELISA kit. The results showed that chickens in the rHVT-005/006-F group displayed a 40% antibody positivity rate at 2 wpv, 90% positivity at 3 wpv, and a 100% positivity rate at 4 wpv with a titer of 7026.0 ± 2370.5 (Figure 3A). In contrast, chickens in the rHVT-US2-F group showed a 20% antibody positive rate at 2 wpv, 80% positivity at 3 wpv, and 90% positivity at 4 wpv with a titer of 5401.1 ± 2455.4 (Figure 3A). Unvaccinated chickens did not produce antibodies at any of the tested time points (2 wpv, 3 wpv, and 4 wpv). Chickens in both the rHVT-005/006-F and rHVT-US2-F immunization groups successfully induced the production of specific neutralizing antibodies (Figure 3B).

**Figure 4:**
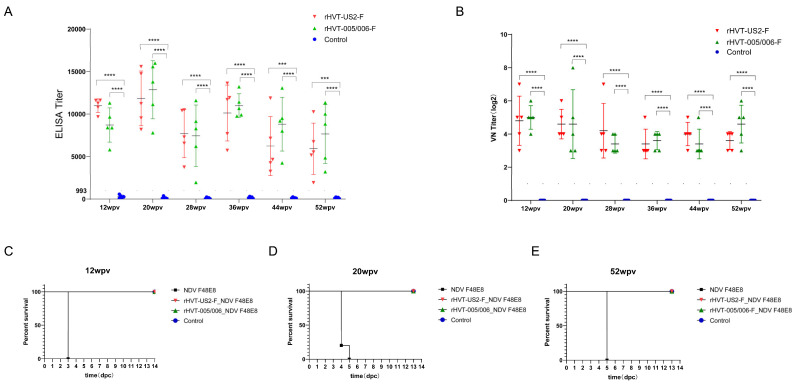
(**A**) Humoral immunity induced by rHVT-005/006-F and rHVT-US2-F. Antibody levels were assessed using a commercial NDV antibody ELISA kit. The y-axis represents antibody titers, with titers ≥933 considered positive. The x-axis represents sera samples were collected at 12, 20, 28, 36, 44, and 52 wpv. (**B**) Neutralizing antibody titers against virulent NDV in SPF chickens inoculated with rHVT-005/006-F and rHVT-US2-F at 12, 20, 28, 36, 44, and 52 wpv. (**C**) Survival curve of chickens challenged with virulent NDV at 12 wpv. (**D**) Survival curve of chickens challenged with virulent NDV at 20 wpv. (**E**) Survival curve of chickens challenged with virulent NDV at 52 wpv. *** *p* < 0.001, and **** *p* < 0.0001.

To assess the duration of the humoral immune response induced by a single immunization with the rHVT-F candidate vaccine, serum samples were collected every eight weeks starting from 12 wpv. The results indicated that ELISA antibody levels against F protein gradually increased, peaking at 20 wpv in chickens immunized with rHVT-US2-F and rHVT-005/006-F. At 20 wpv, the antibody titers were 11,850.2 ± 3218.6 in the rHVT-US2-F immunized group and 12,868.0 ± 3417.9 in the rHVT-005/006-F immunized group. High antibody titers were maintained for up to 52 weeks, with levels at 52 wpv being 5922.3 ± 3010.6 in the rHVT-US2-F group and 7656.8 ± 3471.2 in the rHVT-005/006-F group (Figure 4A). Virus-neutralizing antibodies exhibited a similar trend, with neutralizing antibody titers at 52 wpv being 3.6 ± 0.5 Log_2_ in the rHVT-US2-F-immunized group and 4.6 ± 1.1 Log_2_ in the rHVT-005/006-F-immunized group. These results suggest that both rHVT-US2-F and rHVT-005/006-F vaccines could induce high levels of humoral immunity for at least 52 weeks. (Figure 4B)

### 3.4. Protective Efficacy of rHVT-005/006-F and rHVT-US2-F in Chickens

On the 3 dpc, only the challenged chickens without vaccination showed hemorrhage in the proventriculus and cecum tonsils, while no visible lesions were observed in the rHVT-005/006-F- and rHVT-US2-F-vaccinated groups (Figure S3). Pathological examination revealed that only the challenge group displayed congestion and hemorrhage in the intrinsic layer of the proventriculus; extensive necrosis in the spleen with localized reduction in lymphocytes; atrophy of lymphoid follicles; and a reduction in lymphocytes in the cecal tonsils. No lesions were observed in the tissues of the rHVT-005/006-F- or rHVT-US2-F-immunized groups (Figure S4). NDV was not detected in the chickens immunized with rHVT-005/006-F and rHVT-US2-F vaccines, while it was detected in all chickens in the challenged group, which exhibited a higher viral load in the cecum tonsils at 3.16 × 10^7^ ± 0 TCID_50_/100 mg (Figure 3D). Following the challenge, all chickens in the challenged group displayed acute severe clinical disease and died within 4 days, with a mean death time (MDT) of 4.0 days (Figure 3C). Chickens immunized with rHVT-005/006-F or rHVT-US2-F vaccines did not show any clinical symptoms (Figure 3C; Table 2). The same results were found at 12, 20, and 52 weeks (Figure 4C–E; Table 2).

Viral shedding was quantified by inoculating 9-day-old chicken embryos and performing a hemagglutination assay from oral and cloacal swab samples. At 4 wpv, results showed that all chickens in the challenge group shed the virus at 3 dpc and died at 4 dpc. Chickens from rHVT-005/006-F- and rHVT-US2-F-vaccinated groups did not shed the virus at 3, 5, 7, and 10 dpc (Table 2). These results demonstrated that rHVT-005/006-F and rHVT-US2-F could provide complete protection against virulent NDV at 4 wpv. Only one chicken (20%) in the rHVT-005/006-F-immunized group shed the virus on the 5 dpc, while no chickens in the rHVT-US2-F immunized group were found at 12 wpv (Table 2). Interestingly, 40% of the chickens challenged with NDV at 52 wpv were detected to show virus shedding on the 5 dpc and 7 dpc in the rHVT-US2-F-immunized group, but no viral shedding was detected in the rHVT-005/006-F-immunized group (Table 2). Overall, these results indicate that a single-dose immunization with either the rHVT-005/006-F or rHVT-US2-F vaccine can effectively provide clinical protection against the virulent NDV for at least 52 weeks.

## 4. Discussion

ND is a significant infectious disease impacting the poultry industry, characterized by high incidence and mortality, and is classified as a notifiable disease by the World Organisation for Animal Health (WOAH). Epidemiological studies indicate that most ND outbreaks in domestic chickens are associated with genotype VII NDV [9]. Currently, the main vaccines used for ND control are inactivated and live vaccines, which require multiple boosters for layer and breeder chickens, thereby increasing stress-related risks. Several HVT-ND vector vaccines have been launched on the international market [33]. These vaccines offer good cross-protection against various genotypes of ND [18], exhibit some resistance to interference from maternal antibodies [34], and can provide extended protection against ND with just a single vaccination [35]. Vectormune ND and Innovax ND are representative commercialized HVT-ND vaccines [18,22]. These vaccines express the F protein of the D26 strain of NDV (genotype I) at the UL45/46 site or the F protein of the Clone 30 strain (genotype II) at the US2 site. However, due to the antigenic differences between the F proteins of these strains and the currently prevalent virulent strains (genotype XII or VII, etc.) [23,24], there has been a focus on developing recombinant HVT vaccines targeting prevalent virulent strains. For instance, Jia et al. constructed an HVT ND vaccine that expresses the F protein of genotype VII NDV at the US2 site, providing a 70% protection rate against homologous NDV strains at 5 wpv [25]. Calderón et al. constructed another HVT ND vaccine that expresses the F protein of the prevalent Peruvian strain (genotype XII) at the UL45/UL46 site, achieving a 100% protection rate against homologous NDV strains at 7 wpv [26]. However, the early protection effect and the long-term protection of both vaccines remain to be clarified.

Due to the expression of the same antigen at different sites possibly leading to different efficacies of recombinant HVT vaccines [15,27,36], two recombinant HVT viruses, rHVT-005/006-F and rHVT-US2-F, were constructed. Both expressed the modified F protein of NDV genotype VII at either the US2 site or HVT-005/006. Animal trial results demonstrated that these two vaccines could provide 100% clinical protection against virulent NDV strains within 4 wpv. Moreover, chickens vaccinated with either rHVT-005/006-F or rHVT-US2-F continued to offer 100% clinical protection against virulent NDV at 52 wpv. Beyond clinical protection, preventing NDV shedding is crucial for reducing the transmission of the virus among poultry populations [37,38]. A study reported [39] that a single administration of the Vectormune ND vaccine in SPF chickens provided a 95% clinical protection rate against genotype VII NDV at 4 wpv, but it did not completely prevent NDV shedding. In contrast, our vaccines successfully completely blocked NDV shedding in both oropharyngeal and cloacal samples at 4 wpv. This result is particularly significant as it demonstrates a marked improvement in preventing the spread of the virus. Notably, chickens vaccinated with rHVT-005/006-F still showed no detectable virus shedding at 52 wpv. In contrast, some chickens immunized with rHVT-US2-F were detected to shed the virus after challenge. Although the US2 site is a commonly used insertion site in commercial HVT-ND vaccines [13], this comparison highlights the superior ability of rHVT-005/006-F to reduce virus shedding at 52 wpv. These results conclusively demonstrate that the F protein is stably and efficiently expressed at the HVT-005/006 site in vivo, suggesting that this site could be a highly promising candidate for developing multivalent HVT vector vaccines.

The selection of foreign genes can significantly impact the immunogenicity of a vaccine. The NDV genome encodes six structural genes, among which the HN and F proteins are viral-neutralizing antigens and major protective antigens [40]. The F protein contributes more to the serum antibody response and protective immunity than the HN protein does [41]. In addition, the F gene has also been shown to stimulate specific cellular immunity as a protective antigen [17]. In experiments where SPF chickens immunized with rHVT-F or rHVT-HN were challenged with virulent NDV at 4 wpv, rHVT-F provided 90% protection, while rHVT-HN provided only 47% protection [6]. Moreover, although vaccines co-expressing the F protein with the HN protein (rHVT-HN-F) have been reported, there is no evidence that rHVT-HN-F is superior to single-component constructs (i.e., HVT-ND-F or HVT-ND-HN) [42,43]. Commercial HVT-ND vaccines [33], such as Vectormune ND and Innovax ND, both select the F gene as a protective antigen. F proteins are synthesized as inactive F0 precursors that must be cleaved by host cell proteases into F1 and F2 subunits. The cleavage of F proteins is a major determinant of NDV virulence [44]. The F protein cleavage site (G/E-K/R-Q-G/E-R↓L) of the avirulent NDV strain is cleaved by a trypsin-like protease, whereas the cleavage site of the virulent NDV strain (R/K-R-Q-R/K-R↓F) is cleaved by the furin protease [45]. The F gene in other rHVT-F vaccines mainly originates from two sources. One is derived from an avirulent vaccine strain with an avirulent cleavage site, which has shown effectiveness in providing significant protection [35]. Another source of the F gene is from a virulent strain. F-gene DNA vaccines with genotype VII, modified at the cleavage site to resemble an avirulent strain, have been reported to provide better immune-protection against virulent NDV compared with DNA vaccines encoding the wild-type F gene [29]. The F gene was also modified by replacing the cleavage site “112RRQRR↓L117” with the cleavage site “112GRQGR↓L117” from the vaccine strain LaSota in our study. Jia et al. engineered the rHVT-NDV-opti F vaccine expressing the genotype VII NDV F gene [25], which induced NDV-specific antibodies with a positivity rate of 40% in the fourth week and of 60% in the fifth week. Additionally, it offered 70% protection against homologous NDV challenge at 5 wpv. Our vaccine candidates appear to be much more effective than that of rHVT-NDV-opti F. It is possible that the modification of the F protein cleavage site could influence immunigenicity, and the altered genetic codes of the F gene could be unsuitable for expression in the recombinant virus.

Humoral immunity generated post-vaccination is crucial for controlling ND. The rHVT-005/006-F and rHVT-US2-F vaccines exhibited nearly 100% positive antibody titers at 4 wpv, inducing humoral immunity in both cases. In contrast, the rHVT-opti-F vaccine constructed by Jia et al. [25] induced only 40% antibody positivity in immunized SPF chickens at 4 wpv. The rHVT-F vaccine developed by Calderón et al. [26] achieved an antibody positivity of 85.7% at 5 wpv in SPF chickens. These results suggest that the two candidate vaccines we constructed appeared to be much better in inducing humoral immunity than others. Despite this, two chickens (2/10) from the rHVT-US2-F immunization group and one chicken (1/10) from the rHVT-005/006-F immunization group did not detect neutralizing antibodies at 4 wpv, and their protection efficacy was still 100% in terms of both clinical and virus shedding. It has been similarly reported [18] that commercial chickens immunized with the rHVT-F vaccine provided considerable protection against NDV, although the chickens showed negative ELISA antibodies at 4 wpv. This indicates that cell-mediated immunity (CMI) may play a significant role in the rHVT-F vaccine’s effectiveness. In terms of long-term protection, all chickens immunized with rHVT-005/006-F or rHVT-US2-F exhibited detectable ELISA and neutralizing antibodies at 12, 20, 28, 36, 44, and 52 wpv. Another study [35] reported that ELISA antibody titers peaked at 10–15 wpv and remained high until 72 weeks, providing sustained protection against challenge with NDV virulent strains. However, positive ELISA antibody titers were not observed until 6 wpv, possibly due to interference from maternal antibodies in commercial chickens, leading to a delayed humoral immune response. This deficiency could be mitigated by providing immunization with live attenuated vaccines early on [46].

HVT is a cell-associated virus, and it is less influenced by maternal antibodies. Notably, the rHVT-IBD vaccine, which is resistant to maternal antibodies, has been widely used on poultry farms worldwide [47]. We have demonstrated that the vaccine provides good protective efficacy in SPF chickens. Future studies will investigate the extent of interference from rHVT-F in commercial chicken farms. In addition, the route of challenge is crucial as mucosal routes of infection more closely mimic the natural route of infection [39]. Therefore, our current focus is on investigating the local immune mechanisms induced by rHVT-F. For countries where NDV is prevalent, a protection leak for four weeks may be not good for practice. Thus, early booster immunization with attenuated live vaccines, such as the B1 strain [46,48], or the immunization of 18-day-old chicken embryos, may offer more effective early protection. Additionally, since HVT is used as a vaccine strain for Marek’s disease (MD), it is essential to consider its protective effect against MD. Both rHVT and HVT exhibit similar protective effects against MD, but neither provides effective protection against vvMDV (very virulent Marek’s disease virus). It is noteworthy that studies have revealed that co-immunization with rHVT and the MDV serotype I vaccine strain CVI988 offers better protection against vvMDV [13]. This approach can be adopted in clinical applications. Despite this, our results have demonstrated the good immunogenicity of the HVT-005/006-F and HVT-US2-F vaccines, showing great potential for commercialization. We will continue to thoroughly evaluate their performance, aiming to provide more effective protection strategies for the poultry industry.

## 5. Conclusions

Two Newcastle disease vaccine candidates, rHVT-005/006-F and rHVT-US2-F, were developed through the CRISPR/Cas9-mediated NHEJ repair pathway and Cre/LoxP recombination. SPF chickens immunized with either vaccine at one day old achieved 100% protection four weeks post-vaccination, and showed no clinical signs or virus shedding when these birds were challenged with the virulent NDV F48E8 strain. Additionally, a single dose of either vaccine provided clinical protection against NDV for at least 52 weeks. The study also confirms that the HVT-005/006 site can be used as a novel insertion site for constructing multivalent HVT vector vaccines.

## Figures and Tables

**Table 1:** Primers for gRNA/Cas9 plasmid cloning, donor plasmid construction, and identification of recombinant viruses.

Primer Name	Sequence (5′-3′)	Product Size
NotI-NDV F-F	ATTTGCGGCCGCATGGGCTCCAAACTTTCTACCA	1686 bp
NotI-NDV F-R	ATTTGCGGCCGCTCATGCTCTTGTAGTGGCTCTCA	
HVT-US2-F	ATGGGTGTGTGCATGATAACT	142 bp (HVT) or 3521 bp (rHVT-US2-F)
HVT-US2-R	GCACACCCACATCATTCTTCA	
HVT-005/006-F	TCGTTTGCGCGTAGTAACATT	347 bp (HVT) or 3726 bp (rHVT-005/006-F)
HVT-005/006-R	TAACTGTGAGCAATGCAGGGG	
sgA-F	CACCGAGATCGAGTGCCGCATCAC	Not applicable
sgA-R	AAACGTGATGCGGCACTCGATCTC	
HVTUS2-sg1-F	CACCGTGACGCTGCCTCCACCCTA	
HVTUS2-sg1-R	AAACTAGGGTGGAGGCAGCGTCAC	
HVT005/006-sg2-F	CACCGCATATACTGAATCGTAGGG	
HVT005/006-sg2-R	AAACCCCTACGATTCAGTATATGC	

**Table 2:** Protective efficacy against virulent NDV at different time points post-vaccination.

Challenge Period	Group	Virus Shedding				Incidence Rate	Mortality Rate
		3 dpc	5 dpc	7 dpc	10 dpc		
4 wpv	Control	0/10	0/10	0/10	0/10	0%	0%
	NDV F48E8	8/8	-	-	-	100%	100%
	rHVT-005/006-F	0/10	0/10	0/10	0/10	0%	0%
	rHVT-US2-F	0/10	0/10	0/10	0/10	0%	0%
12 wpv	Control	0/5	0/5	0/5	0/5	0%	0%
	NDV F48E8	5/5	-	-	-	100%	100%
	rHVT-005/006-F	0/5	1/5	0/5	0/5	0%	0%
	rHVT-US2-F	0/5	0/5	0/5	0/5	0%	0%
20 wpv	Control	0/5	0/5	0/5	0/5	0%	0%
	NDV F48E8	5/5	-	-	-	100%	100%
	rHVT-005/006-F	0/5	0/5	0/5	0/5	0%	0%
	rHVT-US2-F	0/5	0/5	0/5	0/10	0%	0%
52 wpv	Control	0/5	0/5	0/5	0/5	0%	0%
	NDV F48E8	5/5	-	-	-	100%	100%
	rHVT-005/006-F	0/5	0/5	0/5	0/5	0%	0%
	rHVT-US2-F	0/5	2/5	2/5	0/5	0%	0%

## Data Availability

Data are contained within the article and Supplementary Materials.

## Supplementary Materials

The following supporting information can be downloaded at: https://www.mdpi.com/article/10.3390/vaccines12060604/s1, Figure S1: Strategy for constructing rHVT-005/-006-F. The schematic illustrates the donor plasmid and knock-in at the HVT-005/006 site. Figure S2: Strategy for constructing rHVT-US2-F. The schematic illustrates the donor plasmid and knock-in at the HVT-US2 site. Figure S3: Lesions in different organs after challenge with virulent NDV. The black arrow indicates the location of the lesion. Figure S4: Histopathological changes in different organs after challenge with virulent NDV. Histopathological changes were observed exclusively in the organs of chickens from the challenge group. Notable findings include substantial infiltration of erythrocytes in the intrinsic layer of the proventriculus (red box, (A)), atrophy of lymphoid follicles and a reduction in lymphocytes in the cecal tonsils (black box, (B)), and extensive depletion and necrosis of lymphocytes in the spleen (blue box, (C)). No apparent histological alterations were detected 1n the other groups.
