# Supplementary material for: Long-Term Protection against Virulent Newcastle Disease Virus (NDV) in Chickens Immunized with a Single Dose of Recombinant Turkey Herpesvirus Expressing NDV F Protein
Source: Vaccines (Basel). 2024 May 31;12(6):604. doi: 10.3390/vaccines12060604 (PMC11209589; doi:10.3390/vaccines12060604)
Supplement: Supplementary file 1 [file vaccines-12-00604-s001.zip › vaccines-2993591-supplementary.pdf]

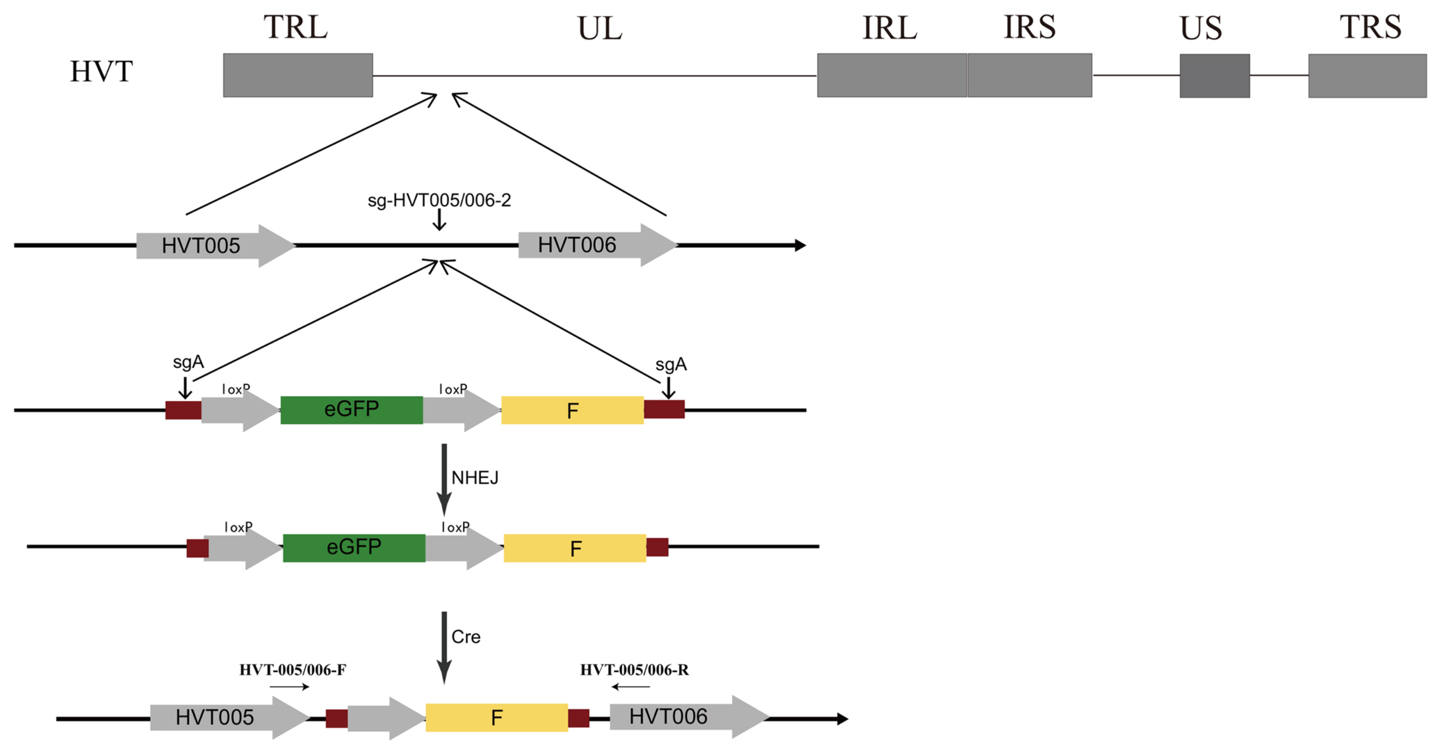

Figure S1 | Strategy for constructing rHVT-005/-006-F. The schematic illustrates the donor plasmid and knock-in at the HVT-005/006 site.

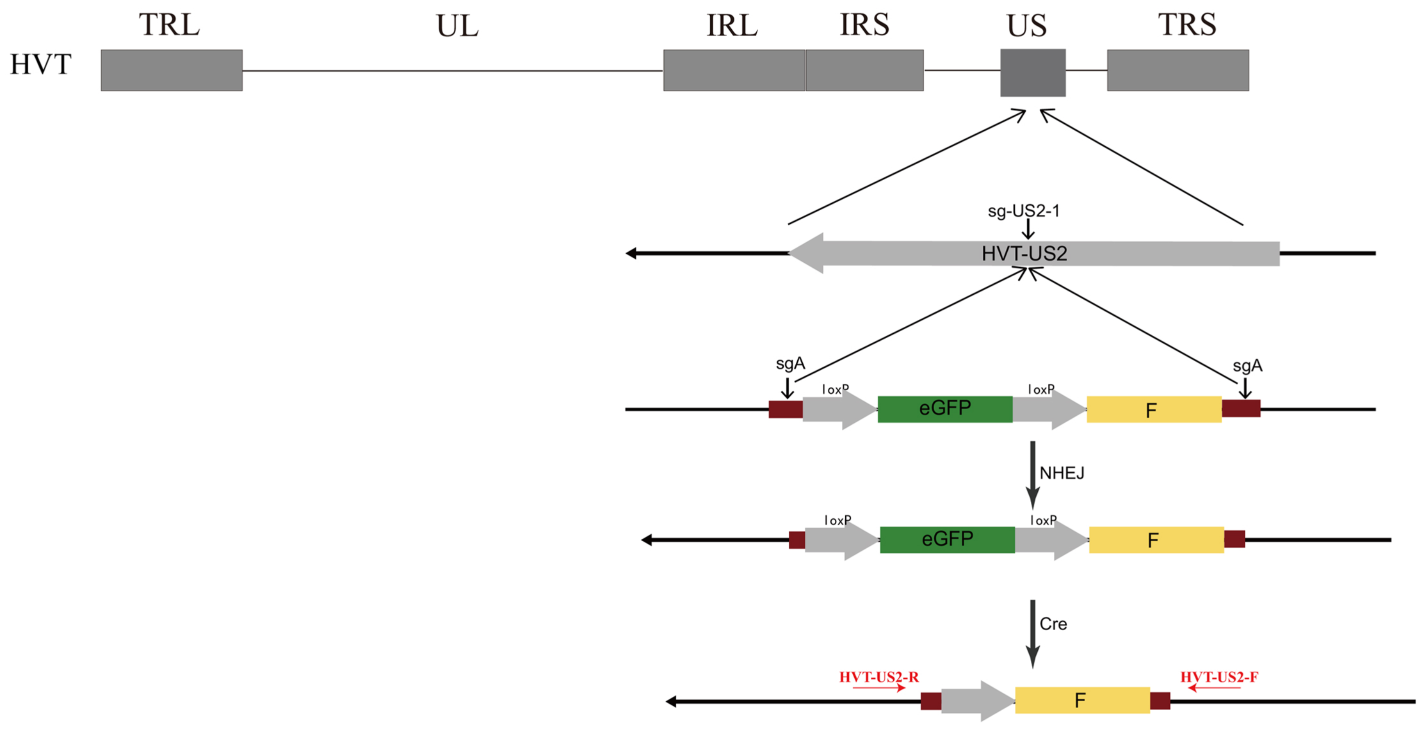

Figure S2 | Strategy for constructing rHVT-US2-F. The schematic illustrates the donor plasmid and knock-in at the HVT-US2 site.

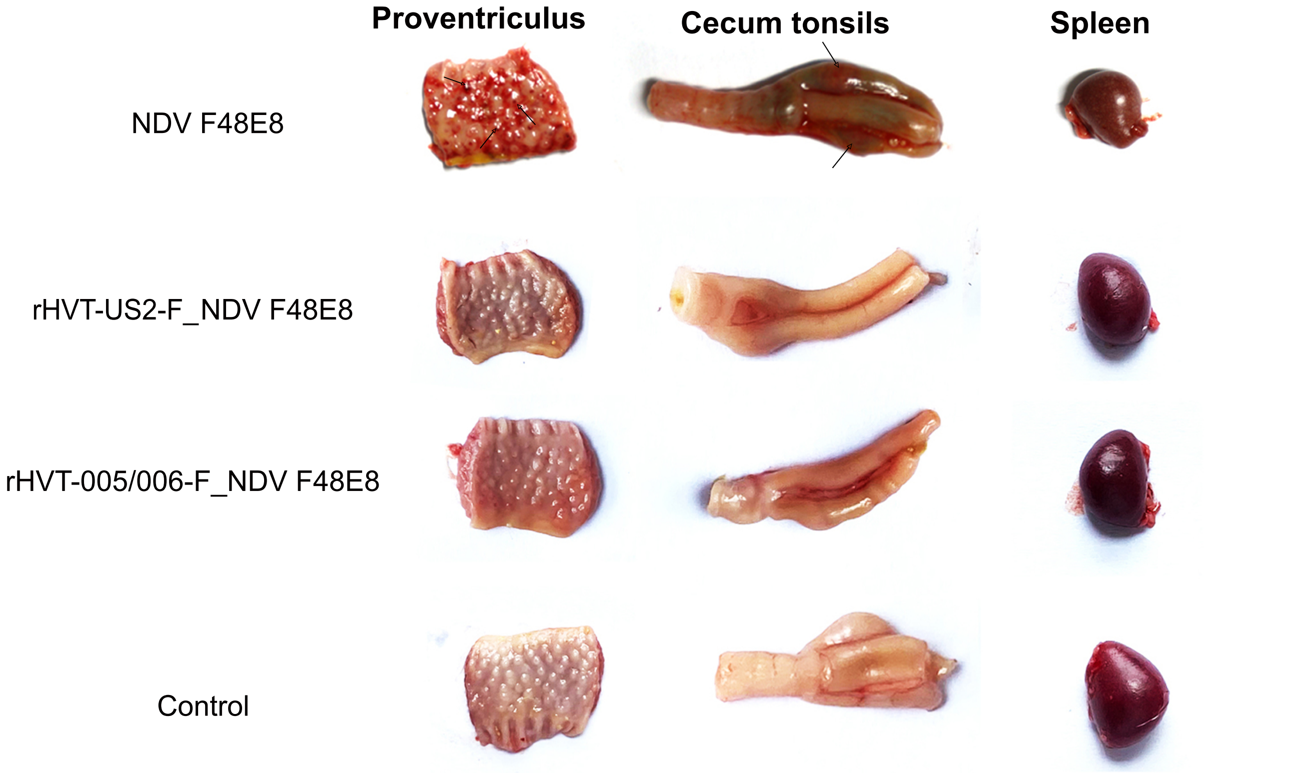

Figure S3 | Lesions in different organs after challenge with virulent NDV. The black arrow indicates the location of the lesion.

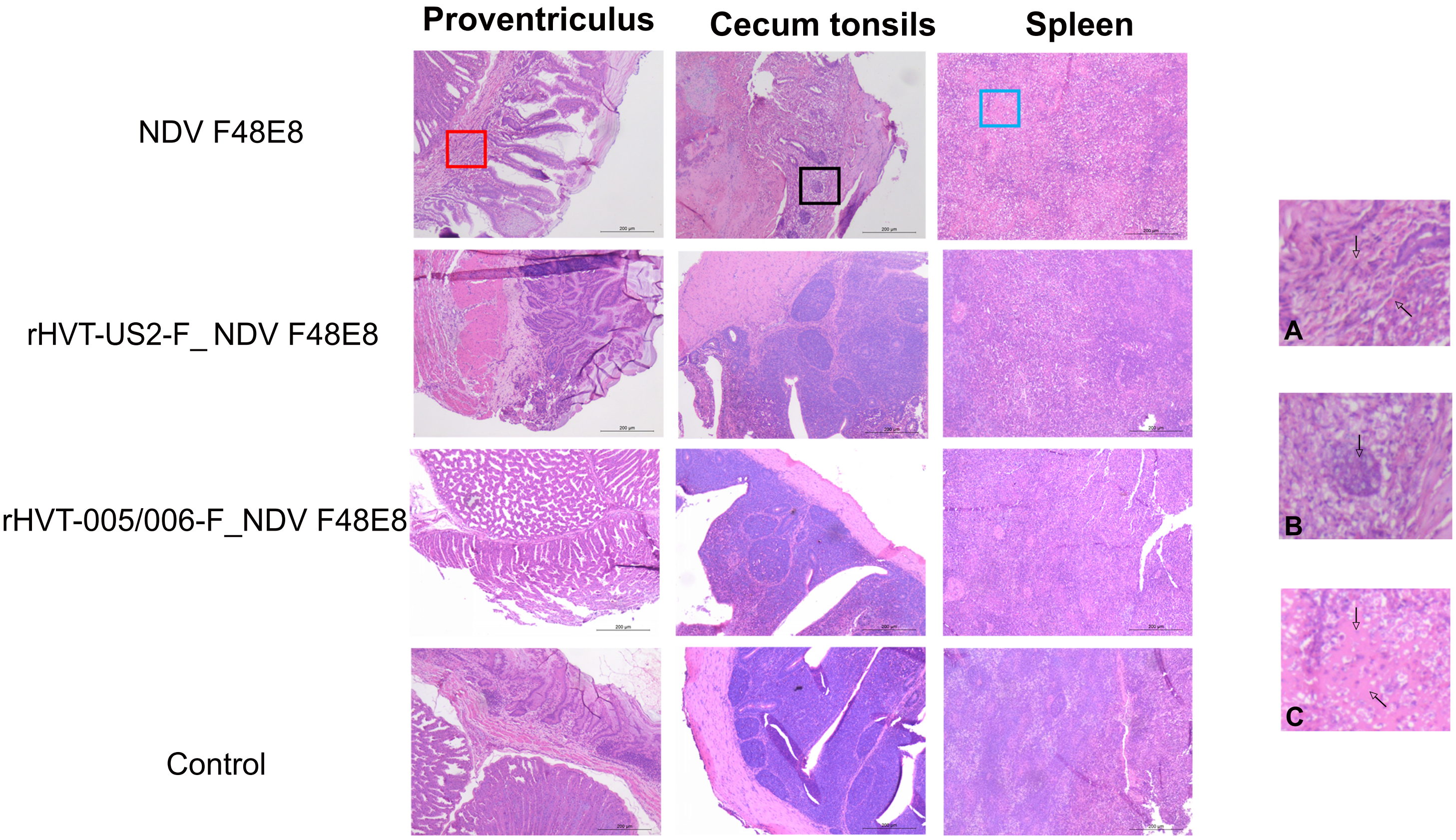

Figure S4 | Histopathological changes in different organs after challenge with virulent NDV. Histopathological changes were observed exclusively in the organs of chickens from the challenge group. Notable findings include substantial infiltration of erythrocytes in the intrinsic layer of the proventriculus (red box, (A)), atrophy of lymphoid follicles and a reduction in lymphocytes in the cecal tonsils (black box, (B)), and extensive depletion and necrosis of lymphocytes in the spleen (blue box, (C)). No apparent histological alterations were detected in the other groups.
